# Thrombotic and Hemorrhagic Complications Following Left Ventricular Assist Device Placement: An Emphasis on Gastrointestinal Bleeding, Stroke, and Pump Thrombosis

**DOI:** 10.7759/cureus.51160

**Published:** 2023-12-27

**Authors:** Joseph Phan, Kareem Elgendi, Masi Javeed, Juan M Aranda, Mustafa M Ahmed, Juan Vilaro, Mohammad Al-Ani, Alex M Parker

**Affiliations:** 1 Internal Medicine, Nova Southeastern University Dr. Kiran C. Patel College of Osteopathic Medicine, Clearwater, USA; 2 Internal Medicine, HCA Healthcare/University of South Florida Morsani College of Medicine, Graduate Medical Education: Bayonet Point Hospital, Hudson, USA; 3 Department of Medicine, Division of Cardiovascular Medicine, University of Florida College of Medicine, Gainesville, USA

**Keywords:** hemorrhagic complications, thrombotic complications, device-related thrombus (drt), stroke, gastrointestinal bleeding, left ventricular assist device, heart failure

## Abstract

The left ventricular assist device (LVAD) is a mechanical circulatory support device that supports the heart failure patient as a bridge to transplant (BTT) or as a destination therapy for those who have other medical comorbidities or complications that disqualify them from meeting transplant criteria. In patients with severe heart failure, LVAD use has extended survival and improved signs and symptoms of cardiac congestion and low cardiac output, such as dyspnea, fatigue, and exercise intolerance. However, these devices are associated with specific hematologic and thrombotic complications. In this manuscript, we review the common hematologic complications of LVADs.

## Introduction and background

Heart failure, a complex clinical condition that results from any structural or functional dysfunction affecting the heart’s ventricles to effectively fill or eject blood, affects over 7 million patients in the United States and carries a significant morbidity and mortality risk [1,2]. Within the last few decades, donor heart availability has limited the total number of transplants performed [3]. LVADs increase survival in patients with advanced-stage systolic heart failure by providing continuous cardiac output, thereby maintaining perfusion and unloading the left ventricle by reducing both blood volume and pressure [4,5]. The device is implanted into the patient’s chest, with an inflow cannula that pulls blood from the left ventricle and an outflow cannula that directs blood to the aorta [6]. HeartMate 3, HM3, the only commercially available LVAD in the United States, works by providing continuous flow at fixed speeds using full magnetic levitation (Full MagLev) flow technology [7]. The use of magnets helps levitate the pump’s rotor system, eliminating the need for mechanical bearings, which cause less wear and tear on the device and reduces the shear of blood that passes through [7]. By providing continuous centrifugal flow at fixed speeds, the LVAD ensures adequate perfusion of vital organs by preserving cardiac output in patients with severe left ventricular dysfunction [8].

The hematologic complications associated with LVADs include gastrointestinal (GI) bleeding, stroke, and pump thrombosis [9]. With each generation of LVAD devices, the hematological adverse event profile often varies. Whilst the Heartmate XVE (Thoratec Corporation, Pleasanton, CA, USA) device was pulsatile, characterized by rhythmic blood flow resembling a natural heartbeat, the second and third-generation devices promote continuous flow physiology through fixed-speed motor promoting constant perfusion [5,9,10]. The Heartmate II device (HMII; Abbott Laboratories; Abbott Park, IL, USA) uses an axial flow rotor, which was then developed into a centrifugal flow device in the HeartWare device (HVAD; HeartWare Corp, Framingham, MA, USA) and Heartmate 3 (HM3; Abbott Laboratories; Abbott Park, IL, USA) [9,11,12]. Each subsequent generation of devices has offered a longer mean survival and duration of support [9,10,12,13]. Currently, the only commercially available LVAD in the United States is the HM3 [13]. However, new devices are being researched [14]. In this manuscript, we describe the pathophysiology, diagnosis, and treatment of the hematologic complications of LVADs, namely GI bleeding, stroke, and pump thrombosis.

This article was previously presented as a meeting poster at the Florida Chapter - American College of Physicians: Residents and Medical Students Spring 2023 Poster Competition on March 25, 2023.

## Review

Gastrointestinal bleeding

Epidemiology

LVAD-associated GI bleeding is the most common complication of LVADs requiring hospital readmission [15]. From the Multicenter Study of MagLev Technology in Patients Undergoing Mechanical Circulatory Support Therapy With HM3 (MOMENTUM 3) trial, the incidence of GI bleeding within 2 years of an HM3 implant was 17.7% (bridge to transplant (BTT) or bridge-to-transplant candidacy (BTC)) and 28.7% (destination therapy (DT)), with HM3 being superior to HMII (Table 1) [9]. The risk for GI bleeds increases with a longer duration of implantation, 21%, 27%, and 31% at 1, 3, and 5 years, respectively [11]. At 60 days post-LVAD implantation, the hospital readmission rate for GI bleeding was significantly higher (8.7% vs 2.3%) than heart failure patients without LVAD [16]. During hospitalization, the chance for mortality can be as high as 11% (Table 1) [17].

**Table 1:** Epidemiology of gastrointestinal bleeding, strokes, and pump thrombosis HMII: Heartmate II; HVAD: HeartWare; HM3: HeartMate III; BTT: bridge to transplant; BTC: bridge-to-transplant candidacy; DT: destination therapy *The 2-year survival rate of HM3 is 85.1%, but no specific mortality rates are reported for gastrointestinal bleeding or pump thrombosis for patients with HM3 [26].

	Incidence	Mortality Rate	Most Prevalent Type
Gastrointestinal Bleeding	BTT/BTC: 17.7% (HM3) DT: 28.7% (HM3) [9]	*11% (HMII; inpatient mortality) [17]	Midgut arteriovenous malformation (29-44%) [17-19]
Strokes (Overall)	BTT/BTC: 8.6% (HM3) DT: 10.7% (HM3) [9]		Ischemic strokes (63%) [20]
Ischemic strokes	BTT/BTC: 5.1% (HM3) DT: 6.0% (HM3) [9]	Mortality of 21.1% (HM3 & HVAD; inpatient mortality) [20]	Cardioembolic [21]
Hemorrhagic strokes	BTT/BTC: 4% (HM3) DT: 5.4% (HM3) [9]	Mortality of 88.8% (HM3 & HVAD; inpatient mortality) [20]	Intraparenchymal hemorrhage (47%) [22-24]
Pump Thrombosis	BTT/BTC: 0.5% (HM3) DT: 1.9% (HM3) [9]	*35.6% (HMII) at 6 months [25]	N/A

Most LVAD-associated GI bleeds are derived from the small intestine and upper GI tract [17]. Arteriovenous malformations (AVM) account for the majority of GI bleeding ranging from 29% to 44% (Table 1) [17-19]. The less common GI bleeding complications include gastritis (11%), hemorrhoids (9%), colon polyps (6%), stomach polyps (6%), diverticulosis (3%), and peptic ulcer disease (3%) [18]. Patients were considered to have LVAD-associated GI bleeding if they fulfilled both the Interagency Registry of Mechanically Assisted Circulatory Support (INTERMACS) criteria for an adverse bleeding event and had one or more of the following clinical manifestations: hematemesis, melena, or hematochezia [19,27,28]. INTERMACS criteria for an adverse bleeding event is met by any incidence of bleeding that requires hospitalization, requires transfusion of packed red blood cells (pRBC), results in a hemoglobin drop of greater than 3 g/dL, requires surgical intervention, requires intravenous (IV) vasoactive agents or results in death [19,27]. Melena (39%) is often the most common presenting symptom, followed by hematochezia (32%) and occult bleeding (24%) [17].

The Utah bleeding risk score is a model used to predict LVAD-associated 3-year GI bleeding rates. The seven risk factors included in this score are age >54 years old, history of coronary artery disease (CAD), chronic kidney disease (CKD), prior bleeding, severe right ventricular dysfunction, low pulmonary arterial pressures (<18 mmHg), and elevated fasting glucose (>107 mg/dL) [19]. Other epidemiological factors associated with GI bleeding include male sex, hypertension, atrial fibrillation, ischemic cardiomyopathy, blood type O, diabetes, patients assigned for LVAD DT, pre-LVAD aspirin use, pre-LVAD proton pump inhibitor use, pre-LVAD warfarin use, and concomitant tricuspid valve repair [18,29-33].

Pathophysiology

**Figure 1:**
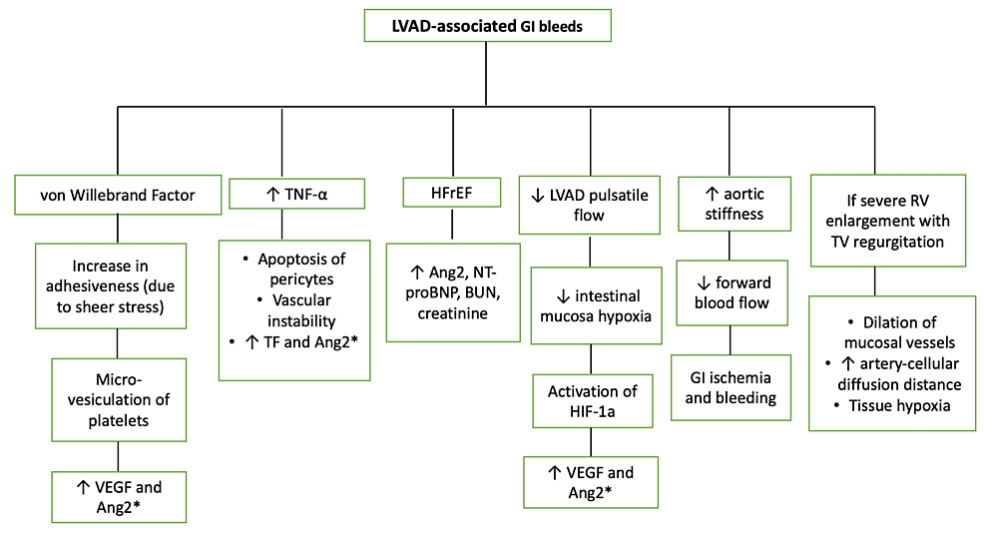
The pathophysiological mechanism of LVAD-associated GI bleeds. This figure highlights the involvement of vWF, increased TNF-α, HFrEF, decreased pulsatile flow, increased aortic stiffness, and severe RV enlargement with TV regurgitation in the development of GI bleeds. Image credits: Joseph Phan and Alex M Parker. GI: gastrointestinal; LVAD: left ventricular assist device; AVM: arteriovenous malformation; TNF-α: tumor necrosis factor-alpha; TF: tissue factor; NT-proBNP: N-terminal-pro hormone brain natriuretic peptide; BUN: blood urea nitrogen; HIF-1a: hypoxia-inducible factor-1a; VEGF: vascular endothelial growth factor. *angiopoietin-2 causes LVAD-associated GI bleeds by disrupting vessel maturation, blocking intracellular connections increasing vascular permeability, and increasing sinusoidal vessel formation.

LVAD-associated GI bleeding may be due to excessive cleavage of von Willebrand factor (vWF) by ADAMTS-13 [34]. The high shear stress of the LVAD device causes a conformational change in vWF to become hyper-adhesive [35]. This activates platelets through micro-vesiculation, which encourages angiogenesis and vascular permeability by releasing vascular endothelial growth factor (VEGF) from platelet alpha-granules and angiopoietin-like protein 2 from endothelial cells (Figure 1) [35-38].

An alternative suggested explanation for LVAD-associated GI bleeding includes elevated serum TNF-alpha which causes angiodysplasia by promoting endothelial proliferation and destabilization through inducing tissue factor expression and pericyte apoptosis, respectively [39]. This results in aberrant angiogenesis due to an imbalance of increased angiopoietin-2 (Ang2) levels from tissue factor expression and decreased angiopoietin-1 levels from pericyte apoptosis (Figure 1) [39]. Additionally, decreased pulsatile flow in LVAD-associated GI bleeding causes intestinal mucosal hypoxia stimulating angiogenesis secondary to the activation of hypoxia-inducible factor 1-alpha (HIF-1a) [40]. HIF-1a increases Ang2, which disrupts vessel maturation, blocks intercellular connections, and forms sinuous vessels that increase the susceptibility to GI bleeding (Figure 1) [28,40]. Increased aortic stiffness after continuous flow LVAD (CF-LVAD) support may contribute to GI bleeding by decreasing the forward flow of effective blood volume into the GI tract, resulting in ischemia and bleeding (Figure 1) [41].

Right ventricular failure likely also contributes to LVAD-associated GI bleeding, specifically AVM-related GI bleeds by dilation of mucosal vessels secondary to a rise in portal venous pressures, increased artery-cellular diffusion distance, and tissue hypoxia (Figure 1) [32]. This suggests the possibility of optimizing hemodynamics before LVAD implantation to decrease the risk of AVM-related GI bleeds [32].

*Assessment and Management *-* Acute GI Bleeding*

As with any acute GI bleed, the European Society of Gastrointestinal Endoscopy (ESGE) guidelines recommend red blood cell transfusion to maintain hemoglobin between 7 and 9 g/dL, blood pressure monitoring, and fluid resuscitation with crystalloid fluids [42,43]. Current medical strategies include temporarily withholding vitamin K antagonists, direct oral anticoagulation, and antiplatelet until hemostasis is achieved for patients with active bleeding [43,44]. However, withholding anticoagulation and antiplatelets should be discussed on a case-by-case basis, considering the balance between bleeding and thrombosis risks [44,45]. For hemodynamically unstable patients, it has been recommended to administer vitamin K with IV prothrombin complex concentrate (PCC) or fresh frozen plasma (FFP) if PCC is unavailable [43,46]. Clinicians must exercise caution when withholding or reversing warfarin because it carries the risk of shifting the patient’s internal normalized ratio (INR) outside the therapeutic range. Additionally, it results in a delay in returning the patient to the desired therapeutic INR levels [47-49]. Having lower than therapeutic INR for a long amount of time may increase the chance of thromboembolic events such as stroke or pump thrombosis [48,49]. High-dose IV proton pump inhibitors, octreotide, and decreasing the speed of the LVAD are other measures to decrease acute GI bleeding [42,45].

For patients hospitalized for LVAD-associated GI bleeding resulting in hemodynamic instability, Axelrad et al. recommend a novel algorithmic approach to endoscopic management [50]. The proposed algorithm necessitates performing push enteroscopy for patients with suspected upper GI bleed (melena, coffee-ground emesis, and hematemesis), colonoscopy for suspected lower GI bleed (hematochezia), and medical management with blood products for occult bleeding (iron deficiency anemia and a positive Hemoccult blood test) [50,51]. For an occult bleed, if more than two units of packed red blood cells are used within 48 hours, push enteroscopy is indicated [50,51]. Adherence to this algorithm increases endoscopic diagnostic (68%) and therapeutic yield (113%), reduction in the number of procedures per patient (18%), decreased length of hospitalization (33%), and reduction of costs (18%) [50].

*Assessment and Management *-​​​​​​​* Long-Term GI Bleeding*

The prophylactic treatment of choice for GI bleeds in LVAD patients is octreotide [52]. Multiple studies demonstrate its efficacy in decreasing the frequency of LVAD-associated GI bleeds, the need for pRBC and PCC/FFP transfusions, and the length of hospitalization [53,54]. Octreotide limits blood pressures in the portal venous system secondary to vasodilation, increasing platelet adhesion, and preventing angiogenesis [17,53].

Danazol is an androgen-like steroid often used to reduce endometrial bleeding and its proposed mechanism to prevent LVAD-associated GI bleeding is through inhibiting endothelial permeability and increasing factor VIII, which is a carrier of vWF [55-57]. Its benefit for treating LVAD-associated GI bleeding has been controversial as a retrospective review published in 2022 showed that danazol had no additional benefit over a lower INR target range approach [58]. An alternative medication, thalidomide, has been shown to decrease the frequency and quantity of LVAD-associated GI bleeding, by inhibiting VEGF, which leads to decreased AVM formation [59,60]. Due to its abundance of adverse effects like dizziness, peripheral neuropathy, bone marrow suppression, hypersensitivity reactions, and pump thrombosis, its use has been limited [53,59,61]. A case series has shown that low-dose thalidomide (daily doses of 200 mg or less) for refractory LVAD-associated GI bleeding is effective and offers a lower overall rate of adverse events [61]. Currently, the distribution of thalidomide is restricted through “thalidomide risk evaluation and mitigation strategies” (REMS) due to the risks of fetal teratogenicity [62].

Digoxin prevents AVM-associated GI bleeding by inhibiting HIF-1a expression [28]. Although continuous digoxin use post-LVAD implantation reduces GI bleeding, its use has not improved the rates of right ventricular failure, mortality, and hospitalization [28,63,64]. The use of angiotensin-converting enzyme inhibitors (ACE-I) and angiotensin receptor blockers (ARB) 30-day post-LVAD implantation has been shown to decrease the incidence of overall GI bleeding [65,66]. These medications work by inhibiting both the transforming growth factor (TGF)- β and the Ang2 pathway [66]. Their efficacy in this scenario is controversial, as one meta-analysis showed no difference in GI bleeding rates compared to the control group [53]. Other potential secondary prophylaxis includes estrogen analogs, doxycycline, desmopressin, bevacizumab, and beta-blockers [53]. Although these medications have mechanisms of action thought to decrease LVAD-associated GI bleeding, their clinical efficacy is conflicting and data supporting their benefit is sparse [28,30,53,65].

Stroke

Epidemiology

From the MOMENTUM 3 trial, patients implanted with an HM3 as a BTT/BTC and DT for 2 years had an overall rate of 8.6% and 10.7% for stroke, respectively [9]. Individual rates of ischemic strokes for patients with HM3 as a BTT/BTC and DT were 5.1% and 6%, respectively [9]. Individual rates of hemorrhagic strokes for patients with HM3 as a BTT/BTC and DT were 4% and 5.4%, respectively [9] (Table 1). Ischemic and hemorrhagic strokes are major adverse events associated with significantly increased inpatient mortality by a factor of 4 and 18, respectively [33]. Furthermore, hemorrhagic stroke has been associated with higher rates of inpatient mortality when compared to ischemic stroke (88.8% vs 21.1%) (Table 1) [20]. This is likely due to comorbidities, fluctuations of anticoagulation use, repeat hemorrhage after resuming anticoagulation, or ischemic strokes when withholding anticoagulation [67]. The most prevalent type of hemorrhagic stroke is intraparenchymal hemorrhage (IPH) followed by subarachnoid (SAH), subdural (SDH), and epidural bleeding [22-24]. Patients with LVAD-associated IPH suffered more neurologic injury and experienced higher 30-day mortality rates than patients with any other type of stroke (38% in IPH vs 0% in SDH, and 29% in SAH) [23].

Associated risk factors for developing either ischemic or hemorrhagic strokes include chronic obstructive pulmonary disease (COPD), hypoalbuminemia, aortic-cross clamping, concomitant cardiac procedure, device thrombosis, post-LVAD infection, hyperlipidemia, history of venous thromboembolism, and previous stroke [68,69]. Modifiable risk factors specific to early and late ischemic stroke include implantable cardioverter defibrillator, pump thrombosis, pump infection, tobacco use, and abnormal coagulation profile (INR <1.6) [33,68,70]. Risk factors specific to early and late hemorrhagic strokes include pump infection, bloodstream infection, hypertension, and prolonged anticoagulation (INR > 3) [68,70]. For LVAD-associated ischemic or hemorrhagic stroke with an initial NIH stroke scale (NIHSS) ≥ 5, a CHIN risk score can be used to predict mortality at 30 days and disability (modified Rankin score ≥4) at 90 days [20]. This score includes risk factors such as creatinine of ≥ 1.5 at stroke onset, hemorrhagic stroke, concurrent infections (must have positive culture), and initial stroke severity using the NIHSS [20].

With the introduction of HM3, the rates of stroke have decreased when compared to HMII, as evidenced by the MOMENTUM 3 trial where the stroke rates for HM3 and HMII were 10.7% and 19.2%, respectively [9,20]. HM3 is favored over HMII due to its ability to enhance 2-year survival without disabling stroke, improve 2-year survival rates, and reduce the incidence of stroke within 2 years [8,9]. In the long term (> 6 months), the incidence of stroke in HM3 was 3.3 times lower than that of HMII [9,71].

Pathophysiology

Cardioembolic events constitute the underlying mechanism responsible for LVAD-associated ischemic strokes [21]. The prothrombotic state is caused by the combination of pump thrombosis, inadequate use of anti-thrombotic, and infections [72]. The pathophysiology of LVAD-associated hemorrhagic strokes is unclear. Some studies attribute its mechanism to endothelial dysfunction and shear stress of the endothelium [22]. In a small case study, acquired von Willebrand disease was associated with intracerebral hemorrhage (ICH) in LVAD patients where five patients with LVAD-related ICH, all tested positive for acquired von Willebrand disease [73]. According to the 2022 Guideline from the American Heart Association and American Stroke Association, ICH is characterized as a type of brain injury that occurs when a ruptured cerebral blood vessel results in the leakage of blood into the brain parenchyma [74]. Active infection and bacteremia are also associated with LVAD-associated ICH [20,75]. The mechanism for this association was infection causing poor platelet function, deficiencies in coagulation factors, drug reactions, bacterial endotoxins, vascular damage, postoperative continuous venous hemodialysis, low serum albumin level, and stress [20,75].

*Assessment and Management *-​​​​​​​* Ischemic Stroke*

As with any type of stroke, LVAD-associated ischemic stroke is diagnosed clinically with the presence of a focal neurological deficit and evidence of infarction on computerized tomography (CT) imaging of the brain [76]. There is no specific guideline for the continuation of anticoagulation during the acute stroke phase [76-79]. However, the risks and benefits should be weighed clinically as anticoagulation with significant infarct size may lead to hemorrhagic transformation.

**Figure 2:**
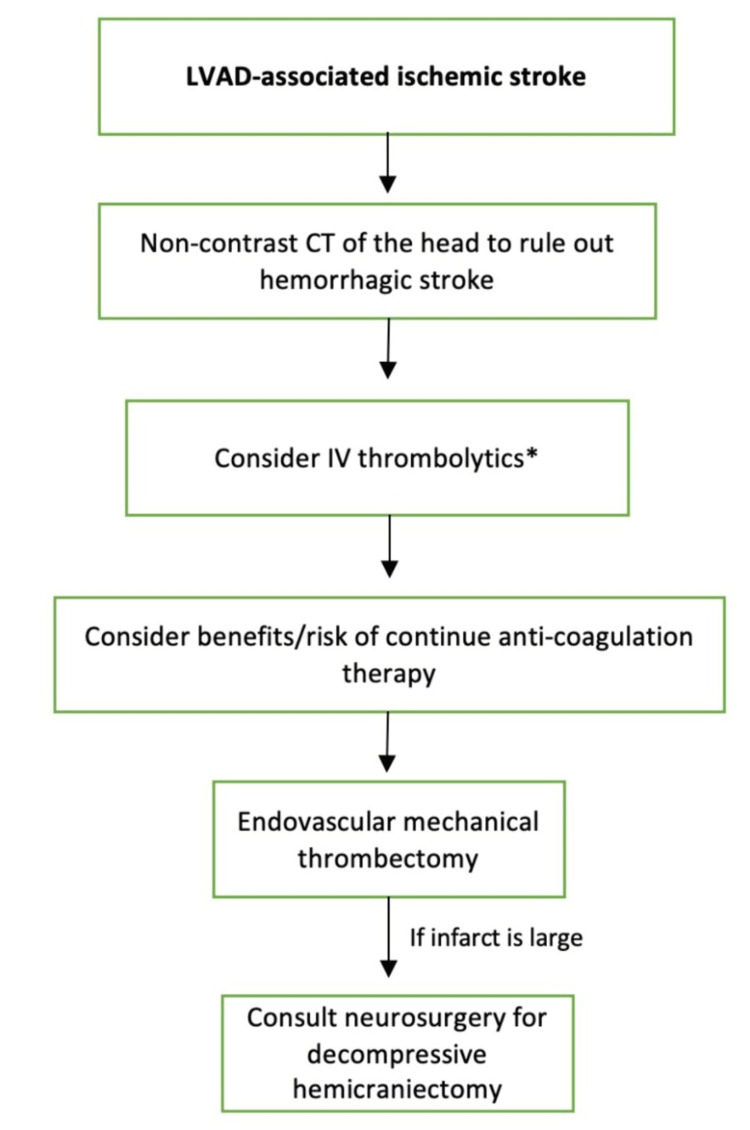
Diagnostics and medical management of LVAD-associated ischemic strokes. Follow up with non-contrast CT of the head to rule out immediate hemorrhagic stroke. The following management includes endovascular mechanical thrombectomy as IV thrombolytics are often contraindicated in patients with LVAD as these patients are on anticoagulation and have a recent bleed. If the ischemic infarct is large, provide cerebral edema management by consulting neurosurgery for decompressive hemicraniectomy. Image credits: Joseph Phan and Alex M Parker. LVAD: left ventricular assist device; CT: computerized tomography; INR: international normalized ratio; aPTT: partial thromboplastin time; HTN: hypertension; IV: intravenous *IV thrombolytics are often contraindicated in patients with LVAD implants

Although IV thrombolytics are often the first line of treatment, it is often contraindicated in patients who have an LVAD due to long-term therapeutic anticoagulation and antiplatelet therapy, delayed presentation, recent major surgery, and recent bleeding events [76]. It is theorized that the thrombi within LVADs are denatured proteins and fibrin that are not responsive to thrombolytics [25]. Therefore, mechanical thrombectomy is the safest and most effective treatment modality because it does not rely on the thrombus composition and minimizes systemic bleeding risk [76]. Unfortunately, mechanical thrombectomy in LVAD-associated ischemic strokes is not without complications as it increases the incidence of intracranial hemorrhage possibly due to acquired von Willebrand syndrome and repetitive device passes to achieve reperfusion [80]. If the infarct size is large, consider the potential need for neurosurgical management of cerebral edema with decompressive craniectomy (Figure 2) [81].

Assessment and Management - Hemorrhagic Stroke

The initial assessment of LVAD-associated hemorrhagic stroke is often a patient showing clinical signs of severe neurologic deficits [21,82]. The initial workup includes non-contrast CT of the head showing hyperintensity suggestive of the hematoma. A CT-angiogram of the head is used to localize the intracranial bleed [82]. Initial medical management implemented by a cohort study in 2020 included discontinuing anticoagulation and reversal of oral anticoagulation using PCC to a goal INR of <1.4 [21,83]. Despite emergency medical management, LVAD-associated hemorrhagic stroke carries a very high risk of mortality [21].

It is recommended to resume anticoagulation with warfarin and antiplatelet therapy after the ICH event to reduce the rate of fatal and non-fatal thrombotic events [23]. The timing to restart anticoagulation and antiplatelet is controversial. Delaying the re-introduction of anticoagulation and antiplatelet agents for up to 30 days is a sensible approach to reduce the risk of recurrent intracranial hemorrhage [83]. However, for individuals at high risk for thromboembolic events, resuming anticoagulation and antiplatelets within 1 week may be required [84]. Platelet transfusions are not recommended with LVAD-associated ICH because it has been linked with hematoma expansion and higher odds of death and dependence at 3 months [23,83]. An exception to platelet transfusions is when planning to undergo neurosurgical intervention [85].

Pump thrombosis

Epidemiology

The reported incidence of suspected or confirmed pump thrombosis in patients with HM3 for BTT/BTC and DT is 0.5% and 1.9%, respectively (Table 1) [9]. From the MOMENTUM 3 trial, HM3 has reduced the rate of pump thrombosis by 12.5% when compared to HMII [86]. Pump thrombosis carries with it significant mortality with a 180-day post-implantation patient mortality two-fold higher than in patients without pump thrombosis (35.6% vs 16.8%) (Table 1) [25].

A multivariable analysis by Grabska et al. identified specific factors that have contributed to pump thrombosis, which included high mean arterial blood pressure, poor anticoagulation and antiplatelet control, and elevated lactate dehydrogenase (LDH) [87]. Cannula malposition and alignment have been associated as risk factors for pump thrombosis due to flow disturbance secondary to deviations of the inflow cannula from the mitral-apical axis [6]. Arrhythmias, such as atrial fibrillation, have been considered as an important modifiable risk for pump thrombosis, due to increased risk for stroke and thromboembolic events [88]. Patients who undergo endocardial radiofrequency ablation experience an approximately two-fold increase rate of pump thrombosis possibly due to the thrombogenic nature of endocardial ablation, which triggers coagulation activation and tissue necrosis [89]. Other rare risk factors, such as heparin-induced thrombocytopenia, have been shown to contribute to an early onset of pump thrombosis [90]. The mechanism is through antibody activation of platelet factor-4 resulting in platelet consumption and thrombosis [90,91].

Pathophysiology

Although several predisposing conditions contribute to the development of pump thrombosis, there is no single underlying pathophysiology to explain the development of pump thrombosis from LVAD use. The pathophysiology of pump thrombosis includes embolus formation due to reduced blood flow, inflow cannula mal-positioning, or insufficient anticoagulation or antiplatelet therapy [92].

A study by Walenga et al. showed that patients with LVAD implantation had elevated levels of inactive protein S, which may be a cause of LVAD-associated pump thrombosis [93]. The study hypothesizes that inflammation derived from LVAD, suggested by elevated C-reactive protein levels, increases the amount of C4bBP, which binds up free protein S [94]. Normally, protein C and its cofactor protein S work to inhibit coagulation factors V and VIII, decreasing thrombin generation [93]. Therefore, the lack of free protein S results in elevated levels of thrombin, fostering a hypercoagulable state favorable for pump thrombosis [93].

Assessment and Management

The criterion used to diagnose pump thrombosis has been compiled by the Interagency Registry for Mechanically Assisted Circulatory Support (INTERMACS), which required at least two of the following parameters: clinical signs of hemolysis in the absence of another etiology, increased LVAD power values (> 30%), isolated elevation of LDH levels >3 times the normal values, and symptoms of newly developed heart failure without another etiology [95]. Other clinical signs and symptoms can aid in the diagnosis of LVAD pump thrombosis, which includes progressive symptoms of heart failure, LVAD low-flow alarm or higher pump power, free hemoglobin > 40 g/dL, and LDH > 800 IU/L [92]. Imaging, such as plain radiographs and CT, can also be used to identify posterior rotation of the inflow cannula, which can be a risk factor for the development of pump thrombosis [92]. By analyzing audio recordings from a patient’s LVAD, which was captured from a digital stethoscope, a recent study showed that LVAD pump thrombosis had an increase in the amplitude of higher-order harmonics, particularly the third harmonic [96]. This detection of acoustic properties may allow for the early detection of pump thrombosis, allowing for the prevention of further complications [96].

**Figure 3:**
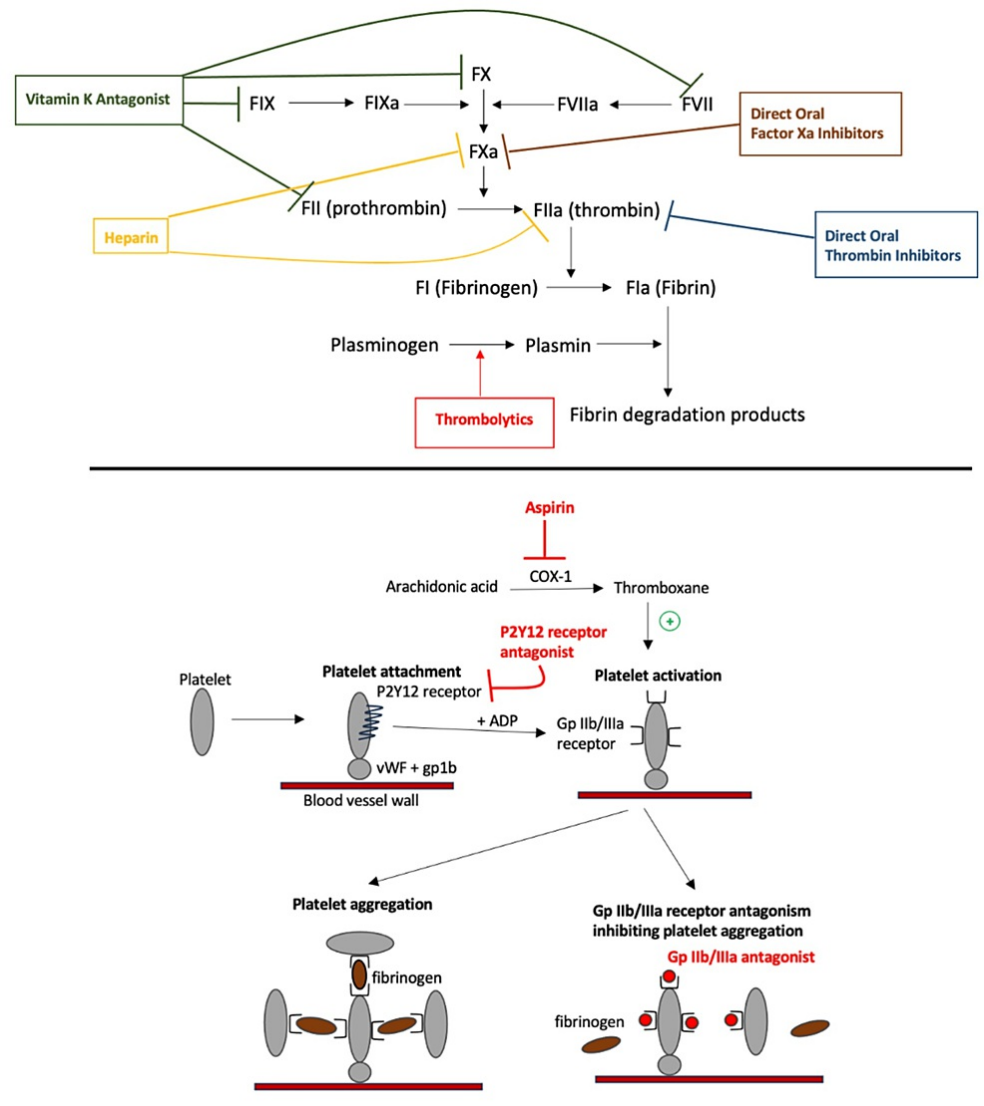
Mechanism of action of various anticoagulants, antiplatelets, and thrombolytics used for prevention or treatment of LVAD pump thrombosis. Anticoagulants include vitamin K antagonists, heparin, direct oral factor Xa inhibitors, and direct oral factor IIa inhibitors. Antiplatelet medications include aspirin, P2Y12 receptor antagonists, and glycoprotein IIb/IIIa inhibitors. Image credits: Joseph Phan and Alex M. Parker. FVII: factor VII; FVIIa: factor VIIa; FIX: factor IX; FIXa = factor IXa; FX: factor X; FXa: factor Xa; FII: factor II; FIIa: factor IIa; FI: factor I; FIa: factor Ia; t-PA: tissue plasminogen activator; vWF: von Willebrand factor; COX-1: cyclooxygenase-1; gp1b: glycoprotein 1b; Gp IIb/IIIa: glycoprotein IIb/IIIa

To prevent pump thrombosis, patients should be placed on both aspirin and warfarin, aiming for a target INR of 2-3, after utilizing a heparin bridge [44]. Aspirin inhibits platelet aggregation by irreversibly inhibiting cyclooxygenase-1 suppressing the production of thromboxane, which amplifies platelet activation (Figure 3) [97]. Vitamin K antagonists, such as warfarin, work by inhibiting vitamin K epoxide reductase, preventing the synthesis of factors VII, IX, X, and II (Figure 3) [98]. The management of pump thrombosis includes either medical therapy with anticoagulation/thrombolytics, pump exchange, or heart transplantation [95]. The initial medical management is IV unfractionated heparin, which acts as an anticoagulant by augmenting the activity of antithrombin III, indirectly inhibiting factor Xa and thrombin (factor IIa) (Figure 3) [95,99]. If hemodynamically stable, but refractory to heparin, tissue plasminogen activator (tPA) may be considered. Thrombolytics, such as tPA, convert plasminogen to plasmin causing thrombin and fibrin breakdown (Figure 3) [95,100]. However, its use for LVAD pump thrombosis carries a risk of major bleeding and hemorrhagic stroke [101]. In addition to anticoagulation, the initial management of pump thrombosis involves administering IV fluids and alkalinization of the urine using sodium bicarbonate drips to protect the kidneys from renal injury secondary to hemolysis of red blood cells [102]. A pump exchange is indicated if the patient is unstable and refractory to heparin [95]. The most definitive therapy for the prevention of LVAD pump thrombosis is heart transplantation if feasible and the patient is deemed an appropriate candidate [92].

The use of glycoprotein (Gp) IIb/IIIa inhibitors, such as eptifibatide, has also been proposed to treat pump thrombosis [103]. These antiplatelet medications block the Gp IIb/IIIa receptor and inhibit platelet binding to fibrinogen, preventing platelet aggregation and thrombus formation (Figure 3) [103,104]. One major limitation of its use is the recorded number of bleeding events [105]. Treatment with these agents may be limited due to the requirement of more aggressive medical therapy, such as fibrinolysis, or device exchange [106]. Additionally, P2Y12 receptor antagonists, such as ticagrelor, in combination with aspirin and heparin, have resulted in symptom improvement, normalization of LDH levels, and decreased need for pump exchange [107]. P2Y12 receptor antagonists cause platelet aggregation by blocking the P2Y12 receptors that normally bind to adenosine diphosphate (ADP), resulting in the decreased expression of Gp IIb/IIIa receptors on platelets (Figure 3) [108]. Evaluating treatment outcomes can be a helpful factor in determining the most suitable management approach for patients with pump thrombosis, particularly when considering the contrast between medical and surgical methods. A systematic review and meta-analysis by Luc et al. showed that surgical device exchange resulted in higher success in thrombosis resolution compared with medical management (81.3% vs. 45.4%), as well as, a lower 30-day mortality and recurrence rate [109].

Direct oral anticoagulants

The use of direct oral factor Xa inhibitors in patients with LVADs, such as apixaban and rivaroxaban, may be a viable treatment option when warfarin therapy has failed [110]. They prevent the development of blood blots by blocking factor Xa activity in the coagulation cascade, preventing the conversion of prothrombin to thrombin (Figure 3) [110,111]. One study showed that thrombotic or hemorrhagic complication rates did not differ between warfarin and apixaban or rivaroxaban suggesting its possible use over warfarin [110,112]. Other studies have even found apixaban to have fewer bleeding complications than warfarin [112]. In contrast, a case study of pump thrombosis was reported to be associated with the use of apixaban after failing warfarin therapy [113]. However, the use of alternative direct oral anticoagulants in patients with LVADs, such as dabigatran, which works as a selective reversible thrombin inhibitor, may have reduced effectiveness in preventing thromboembolic events when compared to long-acting vitamin K antagonist, phenprocoumon [Figure 3] [114,115]. Phenprocoumon is not available for use in the United States [116].

Currently, warfarin is the anticoagulant of choice in patients with an LVAD implant [110]. Due to regular monitoring, dose adjustments, and interactions with drugs and foods, and its association with bleeding and thromboembolic events in LVAD patients, direct factor Xa inhibitors may be a promising alternative to warfarin for LVAD anticoagulation [110]. Although more research is required, direct oral anticoagulants show promise for preventing stroke and pump thrombosis in CF-LVAD patients.

## Conclusions

The common hematologic complications following LVAD implantation are GI bleeding, stroke, and pump thrombosis. With the arrival of HM3, the incidence of adverse hematologic events has decreased when compared to older-generation LVADs. This review article highlights recent discoveries in the epidemiology, pathophysiology, diagnosis, and management of hematologic complications following LVAD implantation. A novel LVAD device known as EVAHEART®2 LVAD (EVA2; Evaheart, Inc., Bellaire, TX, USA) is currently in clinical trials. The analysis of the COMPETENCE trial, which evaluates the safety and efficacy of EVA2, will be important in strengthening the understanding of LVAD-associated bleeding and thrombosis. Further studies should verify the efficacy of direct oral anticoagulants and their use in preventing LVAD-associated hematological complications, especially pump thrombosis, and bleeding.
